# AI with agency: a vision for adaptive, efficient, and ethical healthcare

**DOI:** 10.3389/fdgth.2025.1600216

**Published:** 2025-05-07

**Authors:** Vasco Gerardo Hinostroza Fuentes, Hezerul Abdul Karim, Myles Joshua Toledo Tan, Nouar AlDahoul

**Affiliations:** ^1^ Department of Computer and Information Science and Engineering, Herbert Wertheim College of Engineering, University of Florida, Gainesville, FL, United States; ^2^ Centre for Image and Vision Computing, Centre of Excellence for Artificial Intelligence, Faculty of Artificial Intelligence and Engineering, Multimedia University, Cyberjaya, Malaysia; ^3^ Department of Electrical and Computer Engineering, Herbert Wertheim College of Engineering, University of Florida, Gainesville, FL, United States; ^4^ Department of Epidemiology, College of Public Health and Health Professions and College of Medicine, University of Florida, Gainesville, FL, United States; ^5^ Biology Program, College of Arts and Sciences, University of St. La Salle, Bacolod, Philippines; ^6^ Department of Natural Sciences, College of Arts and Sciences, University of St. La Salle, Bacolod, Philippines; ^7^ Department of Chemical Engineering, College of Engineering and Technology, University of St. La Salle, Bacolod, Philippines; ^8^ Department of Electronics Engineering, College of Engineering and Technology, University of St. La Salle, Bacolod, Philippines; ^9^ Yo-Vivo Corporation, Bacolod, Philippines; ^10^ Department of Computer Science, Division of Science, New York University Abu Dhabi, Abu Dhabi, United Arab Emirates

**Keywords:** agentic AI, artificial intelligence, clinical decision support systems, electronic health records, healthcare administration, healthcare costs, hospital operations, personalized medicine

## Introduction

The healthcare industry continues to face significant operational challenges in patient care, resource allocation, and administrative processes. For instance, despite spending 16.8% of its gross domestic product on healthcare by 2015, the United States reported higher rates of preventable hospitalizations and lower life expectancy compared to countries that spent nearly half as much (1). In fact, the average life expectancy in the United States was 78.8 years, falling short of the 80.6-year average among OECD countries (1). Moreover, 73.2% of insured adults reported experiencing at least one administrative burden that led them to delay or forgo medical care (2). These inefficiencies stem not only from financial concerns but also from deeply embedded flaws in the administrative and technological infrastructure of the healthcare system. Such persistent inefficiencies highlight a need not merely for automation, but for intelligent, adaptive systems capable of navigating complexity in real time.

A major driver of these challenges is administrative overhead. Healthcare institutions allocate approximately 20% of their budgets to administrative tasks, while American physicians spend around 13% of their work time on similar responsibilities (3). Compounding this issue are fragmented workflows, excessive documentation, and poorly integrated clinical systems, which increase physician burnout and the likelihood of clinical errors (4). For example, some computerized provider order entry systems are not tailored to patient needs, requiring 10% more physical effort than manual order selection (4). Additionally, order sets can become outdated quickly, reducing their clinical value. These challenges underscore the need for a more intelligent system to ease administrative burdens and support effective clinical decision-making. What is needed is not just an AI system that follows static rules, but one that learns, evolves, and operates with autonomy.

Agentic artificial intelligence (AI) offers a promising solution by autonomously managing complex healthcare tasks, reducing human error, and enhancing efficiency (5). Using machine learning (ML) algorithms, agentic AI adapts to real-time healthcare environments (6). Unlike conventional AI, which depends on fixed rules, agentic AI acts on its own to achieve healthcare goals and continuously updates its behavior as new information comes in. It can streamline workflows, enhance diagnostic accuracy, and reduce administrative workload (6, 7). Some agentic AI systems have been shown to lower cognitive workload by up to 52% (4). Predictive models powered by agentic AI can identify patients at risk of disease progression or complications, resulting in fewer hospitalizations, reduced healthcare costs, and better outcomes (8). For instance, AI-based monitoring systems can detect subtle changes in vital signs, predict deterioration, and alert clinicians before critical issues develop, enabling timely intervention. Because agentic AI is goal-driven and adapts over time, it is especially well-suited to handle the complexity of hospital environments and ever-changing patient needs. In addition, agentic AI can optimize hospital resource management by dynamically adjusting staffing, supply distribution, and patient flow based on real-time data (5).

This perspective introduces an agentic AI framework designed to automate, optimize, and personalize medical services. Unlike traditional ML models, agentic AI continuously learns from routine data and adjusts its responses to match evolving healthcare demands. This combination of adaptiveness and autonomy makes agentic AI not just an innovation, but a necessity for the future of healthcare delivery. By reducing administrative burdens, enhancing clinical decision-making, and streamlining operations, agentic AI has the potential to transform healthcare delivery and improve both outcomes and cost-effectiveness.

## Reclaiming time for care: the potential administrative power of agentic AI

AI enhances administrative efficiency by automating routine tasks such as medical documentation, insurance claims processing, patient scheduling, and staff coordination (9, 10). In the United States, healthcare professionals spend approximately 25% of their working hours on administrative duties (9). This administrative burden contributes to physician burnout, which in turn affects the quality of care delivered to patients (11). Automating repetitive and time-consuming administrative tasks can therefore free up valuable time for medical professionals to focus more on direct patient care. Agentic AI builds upon this by not only executing administrative tasks but also proactively refining and restructuring workflows. AI-driven automation addresses this issue by significantly reducing manual workloads, enabling healthcare providers to allocate their time more intelligently and efficiently (12, 13).

One of the most time-consuming administrative tasks is the documentation of clinical history. Physicians often spend more than an hour documenting electronic health records (EHRs) for every hour spent with patients, contributing to both burnout and inefficiencies (14). To address this, AI-powered natural language processing and voice recognition technologies are being integrated into EHR systems to assist with medical transcription (15, 44). These tools allow physicians to dictate patient notes, which are then structured into standardized documents by AI. When these systems use agentic AI, they can learn each clinician's preferences over time to improve documentation quality. This reduces documentation errors, improves the accuracy of patient records, and minimizes delays in administrative processing (16). These innovations also support interdepartmental coordination, improve hospital logistics, and enhance physician productivity (17, 18). In addition, automated documentation tools help ensure regulatory compliance by maintaining records that meet legal and ethical standards, thereby reducing the risk of malpractice claims and institutional penalties (9).

AI also improves the processing of insurance claims by identifying errors, ensuring compliance, and detecting fraudulent activity. Traditional claims processing is often time-consuming, prone to human error, and financially inefficient, leading to delayed reimbursements and financial losses for healthcare providers (19). AI-based systems can analyze large volumes of claims data, flag inconsistencies, and verify compliance with regulations, ultimately reducing administrative workloads (45). These optimizations lead to fewer rejected claims, better cash flow, and improved patient satisfaction due to faster approvals (20). AI also detects suspicious patterns in claims, helping to prevent financial losses from fraud (46). Agentic AI would let these systems spot fraud and errors more accurately by learning from feedback and by adjusting to new billing rules in real time. Thus, by automating insurance processing, AI strengthens the financial health of healthcare institutions and allows clinical staff to redirect their focus toward patient care. Additionally, it improves billing transparency and speeds up reimbursements (21).

In patient scheduling, AI increases efficiency by predicting no-shows, optimizing real-time appointment availability, and adjusting schedules accordingly. One study found that AI-supported reminders reduced no-show rates from 19.3% to 15.9%, which helped ensure timely treatment and better use of provider time (10). Furthermore, agentic AI would go even further by learning from patient behavior and clinic flow changes. It could adjust scheduling priorities and timing with minimal human input. These systems reduce waiting times, minimize administrative delays, and prevent resource wastage, improving the overall delivery of healthcare services. Virtual assistants powered by AI also help patients schedule appointments and handle inquiries, reducing the workload of administrative staff and improving the patient experience (22). These tools promote smoother operations, better coordination between providers and patients, and more effective resource use. Moreover, AI-powered assistants enhance accessibility by helping patients with disabilities or language barriers navigate healthcare services (23).

Finally, AI strengthens staff coordination by optimizing workforce management, automating administrative scheduling tasks, and projecting staffing needs based on real-time patient demand. AI scheduling systems analyze historical data, seasonal patterns, and patient inflow trends to assign personnel efficiently and maintain proper coverage during peak periods (24). This helps prevent employee burnout while ensuring that high-quality patient care is maintained (25). Predictive analytics can also assist administrators in anticipating staffing shortages and reallocating resources dynamically (26). Agentic staff coordination systems would respond to changing demands. It would reallocate staff and update schedules on their own in real time. This helps maintain workforce resilience and ensures effective clinical coverage. By integrating AI into staff coordination, healthcare facilities can enhance workforce efficiency, improve operational resilience, and raise the standard of care. However, it is crucial that AI is implemented with care, so that over-optimization does not result in staff reductions that compromise patient care (27). Agentic systems can be designed to prevent such over-optimizations by balancing administrative efficiency with patient-first policies.

## Precision in practice: how agentic AI can strengthen clinical decisions across the care continuum

The application of AI in Clinical Decision Support Systems (CDSS) has enhanced diagnostic accuracy by reducing medical errors and improving patient outcomes (28). AI-powered CDSS provides clinicians with real-time predictive data, evidence-based recommendations, and risk assessments. One study found that such systems led to a 5% change in treatment decisions, driven by more accurate diagnoses and improved decision-making processes (16). By leveraging large datasets and integrating genetic, lifestyle, and environmental factors, AI enables the automation of personalized treatment plans that enhance patient care (29). With agentic AI, these systems move beyond fixed rules by constantly updating their diagnostic models based on real-world clinical data, making their recommendations more accurate and tailored to current conditions.

One of the most impactful applications of AI in CDSS is diagnostic imaging, where it has surpassed human radiologists in detecting certain diseases (30). AI-powered imaging tools have significantly improved tuberculosis screening in low-resource settings, particularly where access to radiologists is limited (30). AI has also transformed early cancer detection by identifying tumors at earlier, more treatable stages, thereby allowing clinicians to develop timely and individualized treatment strategies (31). In ophthalmology, AI helps detect and grade diabetic retinopathy, increasing access to eye screening and minimizing delays in diagnosis (32). These technologies are especially valuable in regions with limited access to trained medical professionals, offering a cost-effective and scalable solution. In parasitology, for instance, automated diagnostic systems have demonstrated high accuracy in identifying host infection and estimating parasite load, showing particular promise for image-based analysis in low-resource settings (33). Such systems reduce the need for manual interpretation, accelerate diagnostic workflows, and extend effective care to underserved populations. By automating diagnostic processes, agentic AI systems not only facilitate earlier interventions and diagnostic precision but also autonomously learn from each new imaging dataset, which improves its interpretive accuracy over time. However, concerns about interpretability remain. Clinicians must be able to understand and verify AI-generated outputs to ensure the reliability and safety of diagnostic recommendations (13). Agentic AI systems must therefore prioritize explainability to preserve clinician trust and avoid over-reliance on just algorithms. The FUTURE-AI guidelines emphasize that explainability is desirable not only from a technological and clinical perspective, but also from ethical and legal standpoints. They recommend defining explainability needs with end users and implementing human-in-the-loop mechanisms to detect bias, flag implausible outputs, and override AI decisions whenever necessary (34).

Beyond imaging, AI-based CDSS has made substantial contributions in pediatrics, particularly in the early detection of sepsis and other life-threatening conditions. Trained on large datasets, these AI models enhance the accuracy of sepsis detection without increasing false alarms or contributing to alert fatigue (35). AI also holds promise in mental health care by enabling early diagnosis, treatment planning, and personalized therapy recommendations for psychiatric conditions (12). These systems can monitor mood and behavioral trends continuously, supporting timely interventions and improved patient well-being. Agentic AI enables these systems to recalibrate detection thresholds over time as they process behavioral data. This improves the accuracy of mental health interventions and reduces misclassification. However, if the training data lacks demographic diversity, AI may inadvertently reinforce biases in mental health diagnosis, risking misdiagnosis or inadequate care for underrepresented populations (21). Agentic models must therefore have bias-detection and adapt their predictions across varying patient subgroups to ensure fairness and accuracy.

Another important area where AI supports clinical decision-making is in prescription accuracy and drug management. AI algorithms can analyze patient history, genetic information, and drug interaction data to identify potential adverse drug reactions before they occur, thereby reducing medication errors and patient risk (22). In complex cases involving polypharmacy, particularly among elderly or chronically ill patients, AI can detect harmful drug combinations and adjust dosages accordingly. These systems are especially beneficial in resource-limited settings where specialized pharmacists are unavailable (21). By incorporating AI into prescription workflows, clinicians can minimize errors, improve medication adherence, and optimize therapeutic outcomes. Agentic AI would further improve this process by continuously learning from patient outcomes, adapting dosage recommendations, and automatically flagging evolving drug sensitivities.

Finally, AI enhances clinical workflows through integration with EHRs. Machine learning algorithms can evaluate patient histories, lab results, and current health data to identify complications and suggest personalized treatment adjustments (14). In intensive care units, AI-powered monitoring systems continuously assess patient conditions and can predict deterioration before critical events occur (36). In surgical contexts, AI decision support tools provide real-time recommendations based on imaging and individualized risk profiles (14). These developments show that AI in clinical decision support goes beyond diagnostics to enhance treatment precision, workflow efficiency, and overall clinical outcomes. With agentic capabilities, these systems ingest streaming patient data and refine treatment strategies in real time. This high level of adaptiveness is essential for managing acute and rapidly changing conditions. AI also plays a growing role in real-time disease monitoring and adaptive treatment planning (37). Agentic CDSS would bridge the gap between episodic interventions and continuous care by creating personalized treatment paths that evolve alongside changes in patient physiology.

## Efficient by design: the potential operational impact of agentic AI in hospitals

One of the most pressing operational challenges in healthcare is overcrowding in the emergency department (ED). AI offers a solution by predictively analyzing resource needs, using data on historical admission rates, seasonal illness patterns, and external factors such as flu outbreaks and weather conditions (10). It also enables real-time triage by identifying high-risk patients and prioritizing them for immediate care. Agentic AI enhances this process by autonomously refining triage protocols in response to evolving case patterns and treatment outcomes: this means that the system learns from each interaction and improves for future prioritizations. This reduces waiting times for emergency cases and improves overall healthcare operations by enabling hospitals to deliver timely and effective care (35). However, overreliance on AI-based triage systems presents risks, particularly when models fail to recognize rare conditions or when algorithmic bias results in the misclassification of patient severity (27).

Beyond the ED, AI improves hospital-wide workflow efficiency by automating tasks such as discharge planning, bed assignment, and surgical suite scheduling. Agentic AI could expand this functionality by autonomously identifying inefficiencies, learning from its outcomes, and reoptimizing them in real time to balance the changing patient volumes with institutional constraints. AI-driven tracking systems allow for real-time monitoring of patient movement, providing hospitals with a comprehensive view of operational processes rather than isolated data points. This visibility enables administrators to identify bottlenecks and redistribute staff or resources dynamically in response to patient demand (18). AI systems can also forecast departmental overloads and suggest resource reallocation strategies, reducing staff fatigue and optimizing hospital occupancy (46). For example, the YOLOv5s model has demonstrated an effective trade-off between speed and detection accuracy in clinical imaging tasks, making it suitable for real-time applications in resource-constrained environments (38). In surgical departments, AI can support operating room scheduling to allocate teams efficiently, minimize downtime between procedures, and improve surgical throughput (30). When guided by agentic design, scheduling systems can become patient-responsive, balanced with individualized clinical need and real-time prioritization. Despite these benefits, AI-based resource planning must be carefully balanced with ethical considerations to avoid cost-driven decisions that compromise patient care quality (11).

AI also contributes to predictive maintenance of medical equipment. Machine learning algorithms can analyze operational data from devices such as MRI scanners, ventilators, and robotic surgery systems to detect early signs of malfunction. This enables hospitals to schedule maintenance proactively, reducing the risk of equipment failures and costly downtime that can disrupt patient care (45). Predictive maintenance reduces unexpected repairs, lowers operational costs, and improves the availability of life-saving technologies. AI tools can also continuously monitor imaging devices to avoid diagnostic delays, ensuring these tools are always ready for critical use (26). Equipped with agentic AI, these tools can autonomously adapt to usage trends, environmental conditions, and failure probabilities. They could evolve their maintenance schedules without human prompting. However, implementing AI-based maintenance systems requires significant initial investment, which may be challenging for under-resourced healthcare systems (39).

In addition, AI optimizes supply chain operations within healthcare institutions. Predictive analytics enables AI inventory systems to anticipate demand, automate ordering, and prevent supply shortages. During the COVID-19 pandemic, AI-enabled logistics systems played a vital role in efficiently transporting ventilators, vaccines, and personal protective equipment to the areas of greatest need (9). AI-based quality control systems also monitor storage conditions for temperature-sensitive drugs and vaccines, helping to ensure product integrity. Real-time tracking of drug expiration dates prevents overstocking and reduces financial losses due to waste (14). Agentic AI makes supply chains more flexible and responsive by adjusting procurement and distribution based on real-time local patient needs, helping improve care in underserved areas. Although these tools enhance drug safety and reduce costs, improper implementation may widen healthcare disparities by favoring well-resourced institutions over underfunded ones (23).

AI has also transformed surgical operations through the development of robotic-assisted procedures. Systems like the da Vinci Surgical System offer enhanced precision, stability, and visualization for minimally invasive surgeries (12). These systems eliminate hand tremors and allow surgeons to perform complex procedures with greater accuracy. In addition, AI assists with preoperative planning by analyzing patient imaging and health records to develop personalized surgical plans, improving outcomes and reducing postoperative complications (40). AI-enabled surgical robots can also automate routine steps, enabling surgeons to focus on more critical aspects of the procedure (32). Agentic robotic systems further elevate surgical safety and precision by intraoperatively learning from sensor feedback and adjusting their actions dynamically. However, the high cost of robotic surgery limits its accessibility, which may increase disparities in care across institutions (21).

## From cost burden to cost efficiency: economic impacts of agentic AI in health

The application of AI in healthcare has demonstrated substantial economic potential, with estimates suggesting it could contribute between $100 billion and $150 billion annually to the U.S. healthcare system (19, 40). However, these projections largely reflect the impact of conventional AI systems. The integration of agentic AI, capable of autonomous goal pursuit and adaptive learning, unlocks even greater value by allowing healthcare systems to optimize cost structures in real time. These savings arise from improved drug management, reduced hospital readmissions, better resource utilization, and the automation of administrative tasks. By enhancing clinical decision-making and optimizing hospital operations, AI increases healthcare efficiency while lowering unnecessary medical expenditures (9). Agentic AI systems can elevate this further by autonomously reallocating resources and dynamically updating cost-saving strategies based on live institutional data. Thus, agentic AI can go beyond static optimizations to automatically managing costs in response to emerging trends. However, adopting AI requires significant upfront investments in infrastructure, training, and regulatory compliance, which can impose financial strain on smaller healthcare providers (26). The integration of AI can help mitigate these challenges by tailoring resource prioritization to each healthcare institution. In particular, agentic AI can learn from local constraints to develop institution-specific efficiency models that maintain care quality while minimizing operational strain.

One of the primary ways AI reduces costs is through early disease detection and more efficient diagnostics. AI-powered imaging technologies have improved the detection of conditions such as cancer, tuberculosis, and diabetic retinopathy, reducing reliance on invasive and expensive diagnostic procedures (30, 32). Early diagnosis also reduces the need for costly late-stage treatments. Additionally, AI can automate image comparisons and assist radiologists in identifying abnormalities, making the diagnostic process more cost-effective (31). However, disparities in access to AI technologies mean that low-resource institutions may not experience these benefits equally (39, 41). Agentic AI could help bridge this gap by autonomously adapting diagnostic pathways to align with the resources available in low-resource settings.

AI-driven medication management also contributes to cost savings. By analyzing patient history and genetic information, AI can predict adverse drug reactions, which reduces complications and preventable hospitalizations, both of which are major contributors to healthcare costs (21). For chronic conditions such as diabetes and hypertension, AI helps optimize dosages and reduce trial-and-error prescribing, thereby increasing treatment efficacy and reducing waste from ineffective medications (16). Agentic AI extends these benefits by continually learning from patient outcomes and dynamically refining drug regimens to prevent adverse reactions and optimize cost-effective therapies.

Unnecessary procedures and hospital readmissions are significant cost drivers in healthcare. AI helps reduce these by improving diagnostic accuracy and ensuring that appropriate treatment is provided from the beginning. AI-based decision support tools have been shown to lower misdiagnosis rates, leading to fewer redundant tests and imaging procedures (28). For instance, AI can accurately distinguish between benign and malignant tumors, reducing the need for invasive biopsies (46). In cardiology, AI models assess heart disease risk and support early interventions, which can prevent costly emergency procedures (25). Agentic AI further contributes by proactively initiating preventive care workflows and adjusting recommendations as new patient data becomes available, helping institutions avoid costly reactive care.

AI also strengthens hospital supply chain management by improving inventory control and minimizing waste. Predictive analytics can forecast supply demands and maintain optimal inventory levels (15). During the COVID-19 pandemic, AI-driven logistics systems efficiently allocated ventilators, vaccines, and other critical resources to high-need areas (26). AI systems can also monitor storage conditions and track drug expiration dates to prevent overstocking and financial loss due to expired supplies. While these tools support cost reduction, financial optimization should never compromise patient care. Over-automation in budgeting may lead to decisions that prioritize cost over quality, risking negative outcomes for patients (27). A balanced approach is needed to ensure sustainable economic benefits. Agentic AI can help strike this balance by encoding ethical constraints into its optimization logic so that cost-saving measures do not threaten patient outcomes.

Fraud detection and automated billing are additional cost-saving areas supported by AI. These systems can identify suspicious billing patterns, detect duplicate or erroneous claims, and prevent losses due to insurance fraud (9, 20). As a result, hospitals benefit from faster reimbursements and reduced administrative burdens, improving their overall financial health (42). Moreover, agentic systems can improve fraud detection by continuously refining anomaly detection models in response to shifting billing behaviors and fraud techniques.

In the long term, AI contributes to healthcare system sustainability and improves accessibility. AI-enabled telemedicine reduces the need for in-person consultations, lowering transportation and infrastructure costs, especially in rural or underserved areas (39). Additionally, AI-powered chatbots and virtual assistants can handle non-urgent patient inquiries, allowing clinicians to focus on more critical cases (15). With agentic capabilities, these virtual systems could evolve into fully adaptive triage and support agents that could be capable of navigating complex patient needs across multiple healthcare domains.

AI also plays a growing role in drug discovery and clinical trials, helping reduce research and development costs. Traditional drug development is time-consuming and costly, often taking years and billions of dollars. AI accelerates this process by analyzing large datasets to identify promising drug candidates more efficiently (12). Agentic AI enhances this process by iteratively modifying experimental hypotheses and adapting trial designs in real time, thereby reducing the cost of failure and improving discovery pipelines. This contributes to faster access to lifesaving medications and reduces the cost of innovation, making cutting-edge treatments more accessible.

## Discussion and conclusion

Agentic AI offers a fundamentally distinct paradigm from traditional AI by incorporating autonomy, adaptability, and goal-directed behavior into healthcare applications. AI is poised to revolutionize healthcare by enhancing administrative efficiency, improving clinical decision-making, streamlining operations, and supporting economic sustainability. In administration, AI can automate scheduling, documentation, insurance claims, and billing, which reduces the burden on physicians and minimizes human error, but these efficiencies are greatly enhanced when AI systems operate with agentic qualities that enable real-time adjustments based on changing priorities and feedback loops. AI-powered Clinical Decision Support Systems can improve diagnostic accuracy through image interpretation, simplify medication management, and predict clinical deterioration, thereby reducing adverse drug events and medical errors. Operationally, AI can streamline hospital resource allocation, enables effective triage, predicts equipment failures, and enhances supply chain logistics to ensure continuous availability of medical resources. When agentic AI powers these tools, they become responsive systems that evolve in tandem with patient, institutional, and epidemiological changes. And economically, AI can reduce healthcare costs by minimizing unnecessary procedures, decreasing drug waste, and improving fraud detection, which would support better financial planning for healthcare systems.

Overall, AI-driven automation has the potential to make healthcare delivery more efficient, precise, and less burdensome for human professionals. By automating repetitive administrative tasks, AI can allow healthcare workers to focus more on direct patient care. Agentic AI adds a layer of resilience to these systems by making them not only automated but continuously optimized in real-world conditions. Agentic AI ensures that such automation remains adaptive even in extenuating circumstances through the optimization of tasks based on clinical priorities. AI-supported clinical decision-making can enhance patient care by delivering real-time, data-driven insights that facilitate early diagnoses, personalize treatments, and improve drug management. Predictive analytics can further maximize operational efficiency by forecasting hospital demand, optimizing patient flow, and enabling proactive resource allocation. The combined effect of these capabilities can support the creation of a more efficient, patient-centered, and financially sustainable healthcare system. With agentic AI at its core, the healthcare system can become an adaptive network that self-improves to meet both patient and provider needs.

Despite its many benefits, the integration of AI into healthcare presents several challenges. Ethical concerns around patient privacy and data security remain a top priority. AI relies on large datasets, which increases the risk of data breaches, unauthorized access, and misuse of sensitive health information. Furthermore, AI algorithms trained on biased datasets can perpetuate disparities in diagnosis and treatment, particularly among minority populations. Compliance with regulations such as the U.S. Health Insurance Portability and Accountability Act (HIPAA) and the European General Data Protection Regulation (GDPR) must be ensured through continuous oversight, transparent development, and rigorous validation of AI models. In addition to regulatory compliance, developers must investigate and address application-specific ethical issues, especially when AI systems may introduce bias or affect vulnerable populations. Establishing clear responsibilities for AI-related errors is also essential, especially when autonomous systems influence high-stakes clinical decisions. The EU's Act, for instance, categorizes all healthcare AI tools as high-risk and mandates strong obligations around safety, transparency, and performance monitoring. Meeting these obligations requires extensive legal oversight, recurring assessments, and continuous model validation to remain compliant. Furthermore, the legal landscape remains unsettled regarding liability for AI-driven clinical harm. When an autonomous system contributes to a misdiagnosis or adverse outcome, it is unclear whether accountability lies with the institution, the developer, or the clinician, which obviously creates a significant ethical and legal dilemma. Another challenge is interoperability. Many healthcare institutions still use legacy EHR systems, making it difficult to integrate AI solutions effectively. Seamless data exchange between AI systems and existing infrastructure will require standardized protocols and investment in technological upgrades. In addition to interoperability issues, hospitals face other technical barriers to deploying agentic AI. These include the need for real-time data processing capabilities, advanced computational infrastructure, and explainability tools that clinicians can trust. Many existing systems are not designed to support continuous learning models or provide transparent reasoning behind recommendations, which slows adoption, particularly in clinical settings where explainability, real-time responsiveness, and interoperability with legacy EHRs are non-negotiable. These requirements often demand low-latency computation, GPU-accelerated processing, and full-stack AI engineering teams, which many hospitals currently lack. A recent return-on-investment (ROI) study of hospital AI implementation estimated the total five-year cost of deploying an AI platform, including software, infrastructure, and IT time, at approximately $1.78 million, resulting in a 451% ROI. Even over a single year, hospitals experienced a 335% ROI, despite reduced time for full benefit realization (43). These figures highlight that although agentic AI systems require significant upfront financial and technical investment, their long-term efficiency gains can justify the cost. However, this level of investment includes more than just software. It requires infrastructure upgrades, clinician training, deployment coordination across departments, and long-term maintenance planning. For under-resourced hospitals, even partial implementation may be out of reach without phased strategies or external support, raising concerns about equitable access and widening gaps between institutions that can afford to adopt AI and those that cannot. However, implementation also requires careful planning, extensive cross-departmental coordination, and months of deployment time, an estimated 160 working days across IT, radiology, and downstream services, before value is fully realized (43). These practical realities must be accounted for in policy and infrastructure planning, especially for health systems operating on tight budgets. It is also critical to ensure that AI enhances rather than replaces human clinical judgment. Overreliance on AI could erode clinical decision-making skills among healthcare workers. AI should be used as a support tool that augments human expertise rather than serving as an autonomous substitute. Additionally, over-dependence on AI may result in reduced human oversight, increasing the risk of harm if the model produces flawed recommendations. Professional validation and the interpretability of AI-generated decisions are necessary to ensure patient safety. Successful deployment of agentic AI in clinical settings also demands robust traceability and risk management strategies. Traceability measures such as continuous logging, auditing, and performance monitoring are vital for the long-term safety and governance of agentic AI. Furthermore, interdisciplinary collaboration is essential: developers should involve clinicians, legal experts, and ethicists at every stage to ensure systems remain clinically useful and socially responsible. Another concern is the potential for AI to widen global healthcare inequalities. High-resource institutions are more likely to benefit from AI advancements, while low-income countries may lack the infrastructure and technical capacity to implement such systems. Errors in AI models, such as misdiagnoses or inappropriate treatment recommendations, can also cause serious patient harm. These tools must undergo thorough clinical validation and be continuously tested across diverse patient populations to ensure safety and reliability. Agentic AI addresses many of these challenges by continuously adapting to new data and by the possibility of incorporating robust ethical guidelines into its autonomous decision-making processes. What sets agentic AI apart is its potential to dynamically enforce ethical constraints and fairness mechanisms at runtime. This allows healthcare to be safer and more equitable.

To build trust and improve the usability of AI, models must become more transparent and interpretable. Explainable AI (XAI) can help clinicians understand and evaluate AI-generated recommendations. Explainability must be addressed from technological, clinical, legal, and ethical perspectives, ensuring clinicians can scrutinize both AI outputs and their underlying logic. This becomes especially important in agentic AI, where systems are not merely generating suggestions but are independently making context-sensitive decisions that require clinical oversight. In the context of agentic AI, explainability is imperative since clinicians have to be able to scrutinize not only the outcomes but also the autonomous decision-making processes that drive the recommendations. AI also holds the potential to personalize treatments by integrating genetic, lifestyle, and environmental data into care plans. This approach is especially promising for chronic disease and oncology management, where personalized interventions can significantly improve outcomes. The increasing use of Internet of Things (IoT) devices and wearable technologies makes continuous health monitoring possible, allowing AI to track patient data in real time. These insights support early disease detection, enable timely interventions, and improve chronic disease management.

Through the use of agentic AI, these systems would not only monitor patient data but also autonomously adjust treatment recommendations in response to real-time health changes. This adaptivity transforms healthcare from a reactive model into a dynamic, self-learning ecosystem. Thus, the emergence of agentic AI represents a revolutionary leap in healthcare technology that bridges the gap between reactive and proactive healthcare models. It is capable of continuously learning and adapting: making it indispensable for addressing the ever-changing nature of healthcare. To fully harness the potential of agentic AI, stakeholders must invest in robust infrastructure, interdisciplinary collaborations, and ongoing research to ensure these systems remain transparent, ethical, and effective. Furthermore, regulatory standards must evolve to support the integration of agentic AI: these systems should only augment human expertise rather than replace it. By embracing agentic AI, healthcare can transition toward a system that is continuously learning, adapting, and innovating to deliver high-quality and equitable patient care.

In summary, agentic AI is not simply another tool for healthcare automation. It represents a leap toward adaptive, self-improving, ethical, and patient-care-oriented systems. As such, future research should focus on ensuring that AI becomes accessible across a wide range of healthcare settings, including those with limited resources. Low-cost, scalable, and ethically designed AI models are essential for equitable global adoption. While traditional AI has already delivered clear improvements to healthcare administration, decision-making, and cost-efficiency, its successful integration will depend on balancing automation with human oversight, ensuring ethical use, and delivering high-quality care that is accessible to all. Ultimately, agentic AI stands to become the backbone of a healthcare system that does not just respond to change, but anticipates and shapes it in service of better outcomes for all. Embracing agentic AI offers the promise of a healthcare ecosystem that is continuously learning, adapting, and evolving to meet current and future challenges.
